# Systematic evaluation of levodopa-carbidopa intestinal gel patient-responder characteristics

**DOI:** 10.1038/s41531-017-0040-2

**Published:** 2018-01-24

**Authors:** David G. Standaert, James T. Boyd, Per Odin, Weining Z. Robieson, Jorge Zamudio, Krai Chatamra

**Affiliations:** 1 University of Alabama at Birmingham, Birmingham, AL USA; 2 University of Vermont College of Medicine, Burlington, VT USA; 3 Lund University, Lund, Sweden; 4 AbbVie Inc., North Chicago, IL USA

## Abstract

Levodopa-carbidopa intestinal gel (LCIG, carbidopa-levodopa enteral suspension in the United States) is a treatment option for advanced Parkinson’s disease (PD) patients with motor fluctuations. The objective of this investigation was to identify the baseline characteristics predictive of treatment response, measured by improvement in motor symptom severity, in advanced PD patients treated with LCIG during a 54-week, open-label phase 3 study. Patients with ≥1 h improvement from baseline in “Off” time were categorized as “Responders”; whereas those with <1 h improvement, any worsening, or no post-baseline assessment were “Non-Responders”. A subgroup of Responders with ≥3 h improvement in “Off” time was also examined; this subgroup was identified as “Robust Responders”. Baseline demographics and disease characteristics were analyzed and their predictive relationship to change from baseline in normalized “Off” time was assessed. Out of the 324 patients included in the analysis, 272 (84.0%) were categorized as Responders and 52 (16.0%) were Non-Responders. A majority of patients (65.7%) had ≥3 h improvement in “Off” time. In general, baseline characteristics were similar between Non-responders, Responders, and the subgroup of Robust Responders. A conditional tree-structured regression analysis identified baseline “Off” time as the only factor that had significant effect on Responder and Robust Responder status. The safety profile of LCIG was similar between patient groups. Overall, this analysis showed that 84% of LCIG-treated advanced PD patients had ≥1 h improvement in “Off” time and the number-needed-to-treat to observe one patient responder was 1.19 patients. Notably, Responders and Robust Responders to LCIG were observed across the range of baseline demographics and clinical characteristics examined.

## Drug delivery: predicting patient responsiveness

Continuous intestinal administration of Parkinson’s disease drugs in a gel formulation significantly improves motor symptoms in most patients. To identify patient characteristics that predict a favorable response to levodopa-carbidopa intestinal gel (LCIG), David Standaert at the University of Alabama at Birmingham, US, and colleagues examined the effects of LCIG over 54 weeks on 324 patients with advanced Parkinson’s disease. Eighty four percent of patients showed a positive response to LCIG, but the authors were not able to predict responsiveness to treatment (or the risk of them experiencing an adverse effect) based on the patients’ demographics or disease characteristics. Among the responsive patients, they found that those experiencing longer periods of movement difficulties (“Off” time) at baseline, were the ones that benefited the most from LCIG.

## Introduction

Levodopa is the standard therapy for the treatment of Parkinson’s disease (PD). During early disease stage, PD symptoms are usually well controlled with oral levodopa. However, the long-term use of standard oral forms is associated with the development of disabling motor complications (primarily wearing off and dyskinesias) that are difficult to control with conventional oral therapy and often necessitate a transition to more complex advanced PD therapies.^1–7^

Levodopa-carbidopa intestinal gel (LCIG, carbidopa-levodopa enteral suspension in the United States, CLES) is an established treatment option for advanced PD patients with severe motor fluctuations. LCIG is continuously delivered to the upper intestine via percutaneous gastrojejunostomy (PEG-J) and a portable infusion pump. Previous studies, including the primary analysis of this open-label, phase 3 study, have demonstrated that LCIG reduces the motor fluctuations commonly associated with chronic oral levodopa treatment in advanced PD patients.^8–10^ However, the baseline patient characteristics associated with response to LCIG treatment have not been reported. Understanding the baseline characteristics that predict a favorable response to LCIG therapy would be valuable in selecting an optimal therapy for patients with advanced PD.

This post hoc responder analysis investigated the baseline patient characteristics that are predictive of a clinically meaningful response in “Off” time in advanced PD patients treated with LCIG during a 54-week, open-label phase 3 study.^8^ “Clinically meaningful response to treatment” was defined as having at least 1 h improvement in “Off” time compared to baseline and was derived from the minimal clinically important change determined by Hauser et al.^11^ For patients who prematurely discontinued the study prior to the Week 54 visit, the last post-baseline “Off” time was used to define responder status. Patients who did not have post-baseline PD diary assessments were considered Non-Responders. The overall proportion of patient responders and the time taken to reach responder status to LCIG treatment were also examined. Similar comparisons were conducted for the subgroup of Responders with ≥3 h improvement in “Off” time, a subgroup identified as “Robust Responders.”

## Results

**Fig. 1:**
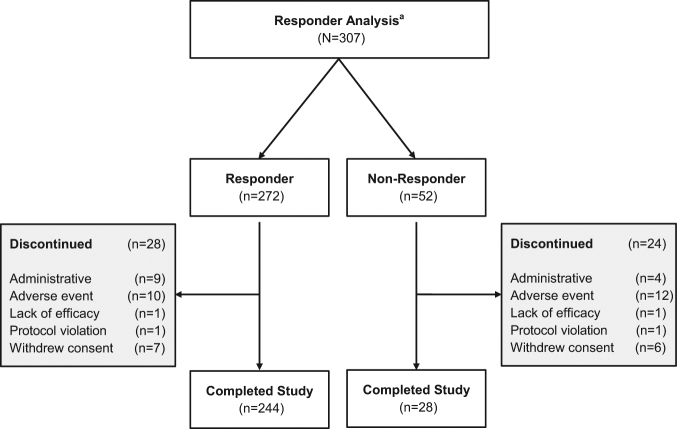
Responder analysis patient disposition. ^a^Patients with a baseline and at least one post-baseline PD diary assessment during the PEG-J treatment phase were included in these analyses

**Fig. 2:**
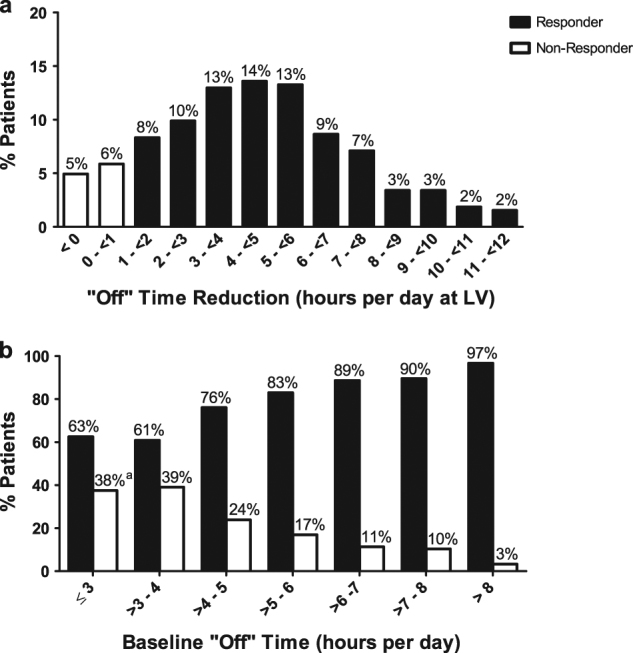
Baseline “Off” time and “Off” time improvement. **a** Percentage of patients with binned hours of reduction in “Off” time from baseline to last visit. **b** Percentage of Responders and Non-Responders by hours of baseline “Off” time. ^a^ Percentage = 37.5%. LV = last visit

Of the 324 patients who had PEG-J placement in the study, 307 patients had post-PD diary assessment, 272 (84.0%) met the criterion for a clinically meaningful response to LCIG treatment in motor symptom severity (at least 1 h reduction in “Off” time at last visit compared to baseline)^11^ and were categorized as Responders (Fig. 1 and Fig. 2). A majority (65.7%, *n* = 213) of these patients had reductions in “Off” time of 3 or more hours compared to baseline (Robust Responders) (Fig. 2a). Of the 272 Responders, 82.4% (*n* = 224) had reached ‘response level’ or responder status (i.e., ≥1 h reduction in “Off” time compared to baseline) by week 4 of LCIG treatment. Fifty-two (16.0%) patients were Non-Responders. Because of the high rate of response to therapy, the number-needed-to-treat in order to observe one patient responder was remarkably low at 1.19 patients.

Baseline patient demographics and disease characteristics were generally similar between the Responder and Non-Responder groups (Table 1). Notable differences between the groups included baseline “Off time”, which was greater for Responders, and “On” time with troublesome dyskinesia (TSD), which was greater for Non-Responders. A similar pattern was observed for patients in the Robust Responder subgroup, which also had similar demographics and disease characteristics compared to the Non-Responder group with the exception of greater hours of “Off” time and less hours of “On” time with TSD at baseline. The proportion of Responders who did not receive any concomitant PD medication other than oral levodopa was 79.8% (*n* = 217) and out of these, 29.4% (*n* = 80) of Responders received LCIG as a monotherapy (no concomitant anti-PD medications). 40.4% (*n* = 21) of Non-Responders did not receive any concomitant PD medication other than oral levodopa and out of these, 19.2% (*n* = 10) received LCIG as a monotherapy.

**Table 1:** Baseline demographics and disease characteristics

Characteristic	Non-responder (*N* = 52)	Responder (*N* = 272)	Robust responder (*N* = 213)
Age, years, mean (SD)	65.0 (9.1)	64.1 (9.0)	64.0 (0.7)
<50 years, *n* (%)	4 (7.7%)	21 (7.7%)	16 (7.5%)
50–64 years, *n* (%)	19 (36.5%)	112 (41.2%)	89 (41.8%)
65–74 years, *n* (%)	22 (42.3%)	110 (40.4%)	87 (40.9%)
≥75 years, *n* (%)	7 (13.5%)	29 (10.7%)	21 (9.9%)
Gender			
Female, *n* (%)	24 (46.2)	115 (42.3%)	87 (40.9%)
Male, *n* (%)	28 (53.8)	157 (57.7%)	126 (59.1%)
BMI, mean (SD)	23.6 (4.3)	25.0 (4.6)	25.1 (4.6)
<25, *n* (%)	37 (71.2%)	146 (53.7%)	114 (53.5%)
≥25, *n* (%)	15 (28.8)	126 (46.3%)	99 (46.5%)
PD duration, years, mean (SD)	13.2 (5.2)	12.3 (5.6)	11.9 (5.6)
<4 years, *n* (%)	1 (1.9%)	8 (2.9%)	8 (3.8%)
≥4 –<7 years, *n* (%)	5 (9.6%)	32 (11.8%)	27 (12.7%)
≥7 –<10 years, *n* (%)	9 (17.3%)	69 (25.4%)	57 (26.8%)
≥10–<15 years, *n* (%)	22 (42.3%)	90 (33.1%)	68 (31.9%)
≥15 –<20 years, *n* (%)	10 (19.2%)	44 (16.2%)	32 (15.0%)
≥20 years, *n* (%)	5 (9.6%)	29 (10.7%)	21 (9.9%)
Modified Hoehn and Yahr Staging, mean (SD)	3.1 (0.8)	3.0 (0.7)	3.1 (0.7)
2.0, *n* (%)	11 (21.2%)	43 (15.8%)	30 (14.1%)
2.5, *n* (%)	4 (7.7%)	40 (14.7%)	30 (14.1%)
3.0, *n* (%)	14 (26.9%)	118 (43.4%)	96 (45.1%)
4.0, *n* (%)	18 (34.6%)	62 (22.8%)	52 (24.4%)
5.0, *n* (%)	1 (1.9%)	5 (1.8%)	2 (0.9%)
Missing, *n* (%)	4 (7.7%)	4 (1.5%)	3 (1.4%)
“Off” time, daily hours, mean (SD)	5.1 (1.7)	7.0 (2.3)	7.5 (2.2)
≤3 h, *n* (%)	3 (5.8%)	5 (1.8%)	0 (0%)
>3–4 h, *n* (%)	9 (17.3%)	14 (5.1%)	4 (1.9%)
>4–5 h, *n* (%)	11 (21.2%)	35 (12.9%)	22 (10.3%)
>5–6 h, *n* (%)	10 (19.2%)	49 (18.0%)	35 (16.4%)
>6–7 h, *n* (%)	5 (9.6%)	39 (14.3%)	32 (15.0%)
>7–8 h, *n* (%)	5 (9.6%)	43 (15.8%)	36 (16.9%)
>8 h, *n* (%)	3 (5.8%)	87 (32.0%)	84 (39.4%)
Missing	6 (11.5%)	0	0
“On” time with TSD, daily hours, mean (SD)	2.4 (2.2)	1.5 (2.0)	1.2 (1.8)
“On” time without TSD, daily hours, mean (SD)	8.5 (2.1)	8.6 (2.1)	7.2 (2.4)
UPDRS total score, mean (SD)	45.1 (21.0)	48.9 (18.6)	50.0 (18.7)
UPDRS Part II score, mean (SD)	17.6 (7.5)	17.4 (6.5)	17.5 (6.3)
UPDRS Part III score, mean (SD)	25.5 (14.0)	29.3 (13.6)	30.1 (13.5)
PDQ-39 Summary Index, mean (SD)	45.0 (16.8)	42.5 (14.7)	42.0 (14.8)
MMSE score, mean (SD)	28.4 (1.9)	28.5 (1.6)	28.5 (1.6)

*BMI* body mass index, MMSE mini-mental state examination, *PD* Parkinson’s disease, *PDQ-39* Parkinson’s disease Questionnaire- 39 item, *TSD* troublesome dyskinesia, *UPDRS* Unified Parkinson’s Disease Rating Scale

Responders to LCIG treatment were observed across the range of baseline demographics and disease characteristics examined in this analysis. A majority of patients were classified as Responders across the full range of baseline “Off” time reported in the study. Out of the 8 patients with ≤3 h of baseline “Off” time, 5 (63%) were Responders and approximately 87 (97%) patients with >8 h of “Off” time at baseline were Responders (Fig. 2b).

**Fig. 3:**
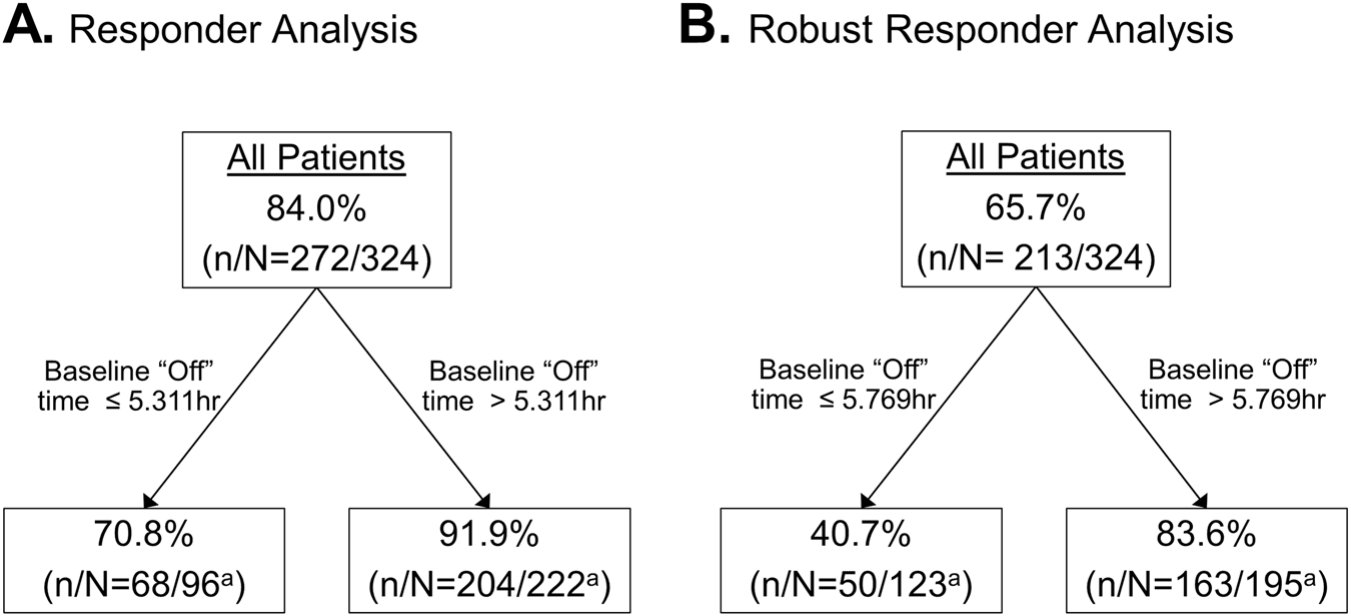
Conditional tree-structured regression analysis of responder rate. **a** Predictive baseline characteristics of Responders. **b** Predictive baseline characteristics of Robust Responders. ^a^Six patients who did not have baseline “Off” time values could not be included

A conditional tree-structured regression method^12^ was applied to evaluate the potential impact of a wide range of variables on the responder status. The following demographic and baseline characteristics were used in the analysis: age, gender, BMI, race, geographic region, PD duration, modified Hoehn and Yahr staging, MMSE total score, “Off” time, “On” time without troublesome dyskinesia, UPDRS Part II score, UPDRS Part III score, UPDRS total score, PDQ-39 summary index, whether the patient experienced treatment adverse event, serious adverse events, severe events, or adverse events leading to premature discontinuation during the study. The analysis showed that baseline “Off” time (with a cutoff of 5.3 h rounded) was the only factor impacting the responder rate (Fig. 3a). Baseline “Off” time (with a cutoff of 5.8 h rounded) was the only influential factor of robust responder rate (Fig. 3b).

### Safety

AEs were reported in 90.4% (*n* = 47) of Non-Responders, 92.3% (*n* = 251) of Responders, and 91.1% of Robust Responders during the entire PEG-J treatment period (Table 2). AE incidences were generally similar between Responder and Non-Responder groups, with the exception of discontinuation due to AE, which was higher for the Non-Responder group (Non-Responders, *n* = 12, 23.1%; Responders, *n* = 10, 3.7%; Robust Responders, *n* = 9, 4.2%). Serious AE and severe AE incidence rates were also higher for the Non-Responder group compared to the Responder and Robust Responder groups (Table 2). The most frequently reported AE in each group was complication of device insertion (Non-Responders, *n* = 20, 38.5%; Responders, *n* = 93, 34.2%; Robust Responders, *n* = 70, 32.9%). Full safety data were previously reported in Fernandez et al., 2015.^8^

**Table 2:** Safety summary

	Number of patients (%)		
Summary	Non-responder (*N* = 52)	Responder (*N* = 272)	Robust responder (*N* = 213)
Discontinued due to:	24 (46.2)	28 (10.3)	20 (9.4)
Administrative	4 (7.7)	9 (3.3)	7 (3.3)
Adverse event	12 (23.1)	10 (3.7)	9 (4.2)
Lack of efficacy	1 (1.9)	1 (0.4)	1 (0.5)
Protocol violation	1 (1.9)	1 (0.4)	1 (0.5)
Withdrew consent	6 (11.5)	7 (2.6)	2 (0.9)
Any adverse event (AE)	47 (90.4)	251 (92.3)	194 (91.1)
Any severe AE	20 (38.5)	76 (27.9)	57 (26.8)
Any serious AE	23 (44.2)	82 (30.1)	64 (30.0)
AEs occurring in ≥10% patients in any patient group			
Complication of device insertion^a^	20 (38.5)	93 (34.2)	70 (32.9)
Abdominal pain	15 (28.8)	86 (31.6)	64 (30.0)
Procedural pain	10 (19.2)	57 (21.0)	49 (23.0)
Nausea	10 (19.2)	44 (16.2)	35 (16.4)
Incision site erythema	10 (19.2)	32 (11.8)	27 (12.7)
Vomiting	8 (15.4)	20 (7.4)	15 (7.0)
Procedural site reaction	7 (13.5)	25 (9.2)	18 (8.5)
Sleep attacks	6 (11.5)	15 (5.5)	12 (5.6)
Pneumoperitoneum	6 (11.5)	13 (4.8)	11 (5.2)
Hallucination	6 (11.5)	10 (3.7)	6 (2.8)
Postoperative wound infection	5 (9.6)	45 (16.5)	33 (15.5)
Constipation	5 (9.6)	42 (15.4)	31 (14.6)
Dyskinesia	5 (9.6)	26 (9.6)	23 (10.8)
Fall	4 (7.7)	45 (16.5)	35 (16.4)
Insomnia	4 (7.7)	40 (14.7)	30 (14.1)
Urinary tract infection	4 (7.7)	33 (12.1)	25 (11.7)
Upper respiratory tract infection	4 (7.7)	4 (1.5)	3 (1.4)
Excessive granulation tissue	2 (3.8)	50 (18.4)	38 (17.8)
Weight decreased	1 (1.9)	30 (11.0)	22 (10.3)

^a^Events with this term were most often additionally coded to abdominal pain, abdominal discomfort, abdominal distension, flatulence, and pneumoperitoneum

## Discussion

The current work represents the first ‘responder’ analysis to investigate the relationship between baseline demographics/disease characteristics and motor response to LCIG treatment in advanced PD patients. This post hoc analysis demonstrated that 84.0% of patients treated with LCIG met the criterion for a clinically meaningful response in motor symptom severity (≥1 h reduction in daily time spent in the “Off” state compared to baseline) and a 65.7% of patients had at least 3 h improvement in “Off” time compared to baseline (Robust Responders). Notably, a majority of Responders reached responder status shortly after LCIG treatment initiation (by week 4). Among the 52 patients that were Non-Responders, 17 did not have post-baseline “Off” time assessment and 35 had <1 h of improvement in “Off” time at the last visit. Twenty-five Non-Responders had reached responder status during at least one time point prior to last visit, making the response rate for patients that reached response level at any time during the study, 91.7% (*N* = 297/324).

Overall, these data support the high rate of response to LCIG treatment observed in previous analyses.^13^ Here, we used a criterion of a 1-h change in “Off” time (compared to baseline) to define Responders, based on previous work by Hauser et al.^11^ This criterion is similar to those used to identify responders in previous studies of the use of rasagiline and entacapone to treat motor fluctuations in levodopa-treated Parkinson disease patients, both of which demonstrated lower rates of response than LCIG.^14,15^ These data also demonstrate a greater responder rate for LCIG than has been previously reported for other advanced PD treatment options, including rotigotine and IPX066.^16, 17^

Baseline patient demographics and disease characteristics were remarkably similar between the Responder and Non-Responder groups. Importantly, Responder and Non-Responder groups had comparable mean disease durations, patient ages, and UPDRS scores. The characteristics of Responders were mirrored by the characteristics of the subgroup of Robust Responders. While significant relationships between LCIG treatment response and most baseline characteristics were largely not observed, the conditional tree regression analysis showed that baseline “Off” time did impact Responder and Robust Responder status in this patient population; patients with >5.3 h of “Off” time at baseline had a greater likelihood of being a Responder. It is important to note that although “Off” time at baseline (>5.3 h rounded) was predictive of Responder status, a majority of patients with few hours of baseline “Off” time (i.e., ≤3 h) were Responders despite having less opportunity for large magnitude of improvement compared to patients with many hours of “Off” time at baseline. Overall, when combined with the high responder rate reported in the current analysis, these data indicate that LCIG was effective for the treatment of motor symptoms in advanced PD patients with a range of baseline demographics and disease characteristics.

The safety profile was comparable between Responders and Non-Responders, with approximately equal numbers of patients in reporting an AE in each group. A similar safety profile was observed for patients in the Robust Responder subgroup. The AE incidence rates were also relatively similar between the groups; however, some AEs including serious AEs, severe AEs, discontinuation due to AE, hallucination, sleep attacks, vomiting, pneumoperitoneum, and incision site erythema, had higher incidence rates in the Non-Responder group (Table 2). Overall the AE profile in each group, including the Robust Responder subgroup, was comparable to the AE incidence rates previously reported in the primary analysis of the phase 3 study.^8^ The safety data analyzed here are limited to the 54 week experience of the study as reported by Fernandez et al., 2015,^8^ and further study of more long-term outcomes will certainly be valuable.

These data provide important clinical information related to LCIG patient selection; however the study design and the analysis do have some limitations. These limitations include the study’s open-label design and the lack of a control group, which does not allow for comparative efficacy and safety assessments. Additionally, this analysis is limited by its post hoc nature; the criterion for the Responder subgroup was guided by the accepted definition of a PD patient responder in the PD literature, whereas the definition of a Robust Responder was guided by expert opinion since there is a lack of information regarding this PD subgroup in the literature.

In summary, this post hoc responder analysis demonstrated that a majority of advanced PD patients treated with LCIG in this study exhibited a clinically meaningful response in “Off” time, whether this was defined by the ≥1 h reduction in “Off” time of the Responder group, or the more stringent ≥3 h reduction in “Off” time of the Robust Responder subgroup. Importantly, these data showed that baseline “Off” time was the only baseline characteristic that significantly impacted Responder and Robust Responder status and that in general LCIG Responders are observed across a range of baseline demographics and clinical presentations. Identifying the patient characteristics that are predictive of positive treatment response is important for selecting an optimal therapy for patients with advanced PD. Data from this responder analysis indicate that the great majority of patients with baseline characteristics that are similar to the eligibility criteria for this study (i.e., levodopa-responsive, having ≥3 h of daily “Off” time at baseline) are likely to have a favorable response to LCIG treatment. Additional research is required to determine the LCIG responder rate in PD patients not meeting this study’s entry criteria, including those patients with more moderate levels of motor fluctuations at baseline.

## Methods

### Study design

This was a post hoc analysis of a phase 3, 54-week, open-label study that evaluated the long-term safety and efficacy of LCIG administered via PEG-J (NCT00335153).^8^ The study protocol was approved by the institutional review board/ethics committee at all 86 centers in 16 countries and performed in accordance with relevant regulations and guidelines. Patients provided written informed consent prior to start of the study.

The study included a screening period (up to 28 days), a nasojejunal (NJ) titration period (2 to 14 days), a PEG-J titration period (2 to 14 days), and a 54-week treatment period. Patients were tapered off any non-levodopa PD medication prior to LCIG initiation with NJ. Patients then underwent a procedure for PEG-J tube placement. LCIG was administered continuously via a portable pump during 16 h of wakefulness. Full study design details were reported in Fernandez et al. (2015).^8^

### Patients

At entry to the study, patients eligible for the phase 3, 54-week, open-label study were ≥30 years of age, had a diagnosis of idiopathic PD according to the United Kingdom Parkinson’s Disease Society Brain Bank criteria, were levodopa-responsive, and had severe motor fluctuations defined as ≥3 h of daily “Off” time despite individually optimized pharmacologic therapy, as judged by the investigator. Patients with an unclear diagnosis of PD, a history of neurosurgical PD treatment, and/or a Mini-Mental State Examination (MMSE) score of <24 were not eligible for study participation.

### Efficacy assessments

#### Pre-specified efficacy analyses

Relevant efficacy outcomes included the mean change, from baseline to last visit, in “Off” time. These outcome measures were derived from a PD symptom diary recorded by patients.^18^ The Unified Parkinson’s Disease Rating Scale (UPDRS) was administered to each patient by the investigator during the patient’s best “On” state. The best “On” time assessments were usually initiated within 2–4 h following the first morning dose of study drug or PD medications. In addition, the 39-item PD Questionnaire (PDQ-39) was used to assess patients’ quality of life. Diary measures and UPDRS and PDQ-39 scores were assessed at weeks 4, 12, 24, and 54 after PEG-J placement; diary measures were also collected at week 36.^18^ Diary variables were normalized to a 16-h waking day and averaged over the 3-days of recordings. Full details related to the pre-specified efficacy analyses of the phase 3 study are reported in Fernandez et al. (2015).^8^

#### Post hoc subgroup analyses

The focus of this post hoc analysis was to analyze the predictive relationship between baseline patient demographics/disease characteristics and change in “Off” time (motor symptom response) after 54-weeks of LCIG treatment.

Patients who had PEG-J placement were included in these analyses (*N* = 324) (Fig. 1). The change in total daily hours of “Off” time from baseline to last visit carried forward was used to stratify patients into groups**:** motor-efficacy Non-Responders (those patients with an improvement from baseline to last visit of less than 1 h, no change, any worsening, or no post baseline assessment) and Responders (those patients with an improvement from baseline to last visit of at least 1 h).^11^ A subgroup of the Responders were identified as Robust Responders (those patients with an improvement from baseline to last visit of at least 3 h). Last visit was define as a patient’s last recorded study visit.

### Safety assessment

Treatment-emergent adverse events (AEs) were monitored throughout the study, coded using the Medical Dictionary for Regulatory Activities (MedDRA) version 14.0,^19^ and tabulated by MedDRA system organ class (SOC) and preferred term. Treatment-emergent adverse events were defined as AEs that began or worsened during the time between NJ tube insertion and 30 days after PEG-J removal. AEs could be coded to more than one preferred term.

### Statistical analysis

The mean change from baseline to patient’s last visit carried forward in daily “Off” time was analyzed. Baseline demographics and disease characteristics were analyzed for each group using descriptive statistics. A conditional tree-structured regression method^12^ was applied to identify the impact of a wide range of variables on the responder status. The following demographic and baseline characteristics were used in the analysis: age, gender, BMI, race, geographic region, PD duration, modified Hoehn and Yahr staging, MMSE total score, “Off” time, “On” time without troublesome dyskinesia, UPDRS Part II score, UPDRS Part III score, UPDRS total score, PDQ-39 summary index, whether the patient experienced treatment adverse event, serious adverse events, severe events, or adverse events leading to premature discontinuation during the study.

### Data availability

Requests for access to data can be made at www.abbvie.com. Primary and secondary outcomes from this clinical trial (NCT00335153) are reported on clinicaltrials.gov at https://clinicaltrials.gov/ct2/show/results/NCT00335153.

### Ethical approval

We, the authors, confirm that we have read the Journal’s position on issues involved in ethical publication and affirm that this work is consistent with those guidelines.
